# Engagement in health and wellness: An online incentive-based program

**DOI:** 10.1016/j.pmedr.2017.05.013

**Published:** 2017-05-18

**Authors:** Teresa B. Gibson, J. Ross Maclean, Ginger S. Carls, Brian J. Moore, Emily D. Ehrlich, Victoria Fener, Jordan Goldberg, Elaine Mechanic, Colin Baigel

**Affiliations:** a Truven Health Analytics, 100 Phoenix Drive, Ann Arbor, MI 48108, United States; b Precision Health Economics, 11100 Santa Monica Blvd. Suite 500, Los Angeles, CA 90025, United States; c Analysis Group, Inc., 1010 El Camino Real, Suite 310, Menlo Park, CA 94025, United States; d Altarum Institute, 3520 Green Ct., Ann Arbor, MI 48105, United States; e stickK.com, 39 East 30th Street, Suite 4, New York, NY 10016

**Keywords:** Wellness programs, Economics, Behavioral, Employer health costs, Online systems

## Abstract

Increasingly, corporate health promotion programs are implementing wellness programs integrating principles of behavioral economics. Employees of a large firm were provided a customized online incentive program to design their own commitments to meet health goals. This study examines patterns of program participation and engagement in health promotion activities. Subjects were US-based employees of a large, nondurable goods manufacturing firm who were enrolled in corporate health benefits in 2010 and 2011. We assessed measures of engagement with the workplace health promotion program (e.g., incentive points earned, weight loss). To further examine behaviors indicating engagement in health promotion activities, we constructed an aggregate, employee-level engagement index. Regression models were employed to assess the association between employee characteristics and the engagement index, and the engagement index and spending. 4220 employees utilized the online program and made 25,716 commitments. Male employees age 18–34 had the highest level of engagement, and male employees age 55–64 had the lowest level of engagement overall. Prior year health status and prior year spending did not show a significant association with the level of engagement with the program (*p* > 0.05). Flexible, incentive-based behavioral health and lifestyle programs may reach the broader workforce including those with chronic conditions and higher levels of health spending.

## Highlights

• Employee engagement with online health promotion activities declined with age.
• No consistent relationship was found between gender and engagement across age groups.
• Employees at all levels of spending, including high spending, used the online program.
• Employees with chronic conditions used the online health promotion program.

## 1 Introduction

Employers have been offering health promotion and wellness programs to employees for decades to boost morale, increase productivity, and address healthcare costs (Employee Benefit Research Institute, 2001, Fries et al., 1993, Glasgow et al., 1995, Jeffery et al., 1993, Ozminkowski et al., 1999, Bertera, 1990, Bly et al., 1986). Most health promotion programs are voluntary and despite their widespread use 61% of employers surveyed noted that poor employee health habits were a key challenge in managing their healthcare costs, the top reason cited (Towers Watson, 2012). While popular among employers, many health promotion programs have suffered from low participation rates (Towers Watson, 2010), low rates of active engagement in health promotion activities (Mattke et al., 2013), and rates of many healthy behaviors among the employed remain low (Hughes et al., 2010). Concerns also exist that many wellness activities are used by healthier employees (Partnership for Prevention, 2008, Thompson et al., 2005), who are more likely to reap benefits, not those with existing chronic illness or high resource use.

Studies have shown that the use of incentives can help improve participation rates in health promotion programs, and field experiments have demonstrated that incentives can help employees lose weight and stop smoking, at least in the short term (Towers Watson, 2010, Volpp et al., 2009, Volpp et al., 2008). Long-term benefits of these programs could also accrue. To further increase the benefits of incentive-based wellness plans some employers are turning to behavioral economics (a field of inquiry focusing on the psychology of economic decision making and behavior such as responses to rewards and incentives) (Ayers, 2010, LDI Issue Brief, 2011) to improve employee engagement in health promotion activities.

In 2014, in the Towers Watson/National Business Group on Health (NBGH) survey of large firms, 69% reported that they offered wellness incentives and the size of the incentives is increasing with time (Towers Watson, 2013). Offering and expanding financial incentives ranked fourth in the top areas of concern for employers with 29% of firms indicating that this was a major area of focus (Towers Watson, 2013).

Despite the adoption of these programs, little is known about actual patterns of use and engagement in health promotion activities reflected in the online program. In this study we define engagement with health promotion activities in a way that is most consistent with the Center for Advancing Health Care's definition, “Actions that people take for their health and to benefit from care.” (Center for Advancing Health, 2010) The goal of this study was to analyze patterns in the level of use of the commitment program and specifically the online incentive program. We compared characteristics of users and nonusers, analyzed individual and aggregate measures of engagement in health promotion activities, and analyzed the utilization patterns of employees to provide one of the first views of real-world use of an incentive-based commitment program with non-trivial financial rewards integrated with information on health status and health spending.

### 1.1 The incentive-based commitment program

In this study, a large nondurable goods US manufacturing firm implemented a customized online commitment program, integrating principles of behavioral economics such as loss aversion (see Table 1 and Ayers, 2010) in the incentive-structure, for workplace health promotion. Each employee could make their own commitments to meet health goals within predefined categories such as ‘Getting Active’ (within this broad category the employee would set their own goal such as 75 min of strength exercise each week for 6 weeks). Employees then earned points that could be redeemed immediately online for gift cards and prizes. Table 1 describes the design of the program.

**Table 1:** Online health promotion program design.

Behavioral economics principle^a^	Program design	Implemented in program
Optimism bias (tendency to believe in positive outcomes)	Encourage precommitment to goals and goal-setting	Commitment contracts were created by employees to meet health goals
Present-based preferences, myopia (focus on present)	Make rewards frequent and immediate for beneficial behavior	⁎ Points were earned for the following activities: enrollment in the program, setting commitments, reporting weekly, use of referee (friend, relative or coworker to validate success), recruiting supporters, success toward meeting health goals, posting online to a commitment journal ⁎ Rewards were selected and redeemed online
Framing and segregating rewards	Employee-selected reward more likely to be effective than a discount on health insurance premiums	Employee-selected rewards: gift cards, sporting event tickets, sweepstakes entries or health-related goods (e.g. pedometers)
Overweighting small probabilities	Provide probabilistic rewards such as a lottery with a larger payoff than employee-selected rewards	Sweepstakes entries were available as a reward
Regret aversion (desire to avoid regret)	Inform of the potential of winning had beneficial behavior been sustained	The largest point allocations were earned at the end of each commitment, and were based on the overall success rate in reaching the health goal (e.g., 75% success toward an exercise goal).
Loss aversion (desire to avoid losses)	Put rewards at risk if behavior doesn't change	Points were not earned if commitment was not successful^b^ Points were deducted if weekly reports not completed
Other incentives	Other program components	Rewards also earned: completing an annual health risk appraisal and completing an in-person biometric screening

a Source of Principles: LDI Issue Brief. Special Issue: Behavioral Economics and Health Annual Symposium. 17(1). September 2011.

b Points could still be earned for setting a commitment, journal entries, recruiting supporters, regular reporting and using a referee.

The first 7500 points earned could be redeemed directly for incentives worth an approximate value of $300 as well as a variety of ongoing sweepstakes with larger dollar values (e.g., personal chef visit or tablet computer) and any points earned above 7500 could only be used to enter sweepstakes. The commitment program was implemented January 1, 2011, coincident with a new comprehensive health and wellness program including biometric screening for risk factors (such as blood cholesterol, weight and blood glucose) and completion of an annual health risk appraisal. To obtain additional incentives, employees were asked to report on progress weekly (for multi-week commitments), to recruit personal supporters, to certify progress with a referee of their own designation, and to make journal entries. Points and rewards were allocated and adjudicated within the online system.

## 2 Materials and methods

We combined detailed 2011 data extracted from the online commitment program with information from 2010 and 2011 administrative medical claims, pharmacy claims, and health plan enrollment data and created measures of program engagement in health promotion activities, health status and resource use, and employee characteristics. When combining administrative data with commitment program data, administrative medical and pharmacy claims, as well as health plan enrollment data were available for users and non-users of the online commitment program, so we could study determinants of use of the commitment program. Participants (users) were defined as employees who signed on to the online program, regardless of whether or not they made a commitment. Sign-on was required to receive incentives offered by the health and wellness program that began at the same time.

### 2.1 Measures of commitment program engagement

Using the data from the online commitment program we collected measures reflecting interactions with the online program: use of the online commitment program, number of commitments made, percentage of commitments where the employee involved a referee for verification, number of online supporters recruited by the employee, number of online journal entries, percent of reports made per commitment, completion of the Health Risk Appraisal (HRA), completion of biometric screening (e.g., blood glucose and Body Mass Index), total points earned in 2011, logon user of the tool (no commitments made but used the tool), completion of a quit smoking commitment with > 50% of reports (typically weekly) indicating that the person did not smoke, and successful completion of a weight loss commitment. These measures reflect the measured program activities of enrollees, and the manner in which enrollees can engage with the online program.

We sought to create a single measure of engagement in health promotion activities by creating a composite of these individual items. First, we evaluated internal consistency of these items (standardized) by examining inter-item correlations, item-total correlations and Cronbach‘s alpha.

We then created a summative scale of the standardized items (equal weight for each item), and, as a comparison, we created a summative score using regression-based factor scores as weights (unequal weight for each item).

### 2.2 Healthcare utilization and resource use

Using healthcare claims, we calculated two measures to reflect health status, and to measure the level of healthcare utilization and resource use in the year prior to program start (2010). The first was a binary indicator of a significant chronic medical or psychiatric illness based on an occurrence of any condition flagged in the Charlson Comorbidity Index (CCI) (D'Hoore et al., 1996, Deyo et al., 1992) (e.g., diabetes) or Psychiatric Diagnostic Groupings (PDG) (Ashcraft et al., 1989) (e.g., depression). The second measure was the amount of total health spending (allowed charges) in the prior year. We analyze these measures to improve understanding of engagement and use of the tool. If we found that the program was being used by high resource users, then those who have more to gain from health promotion activities are using the tool. If, for example, high and low resource users had similar levels of engagement in health promotion activities as reflected by online tool measures, then this suggests that the program is reaching a wide variety of individuals (both high and low resource users).

### 2.3 Employee characteristics

From administrative data we included employee characteristics: age, sex, Census region, health plan, median household income and percent college educated (by ZIP code from the Census files).

### 2.4 Statistical analysis

First, we compared the characteristics of users of the online tool to non-users via a Chi-squared test for categorical variables and a *t*-test for continuous variables. Second, we regressed the engagement index on employee characteristics to determine whether engagement with the tool varied by age, gender, urban area of residence, Census region, area characteristics (median income and percent college graduates), health status (CCI and PDG) and prior year spending. Third, as an exploratory analysis, we regressed 2011 spending measures on the program engagement index and employee characteristics (age, gender, urban area of residence, Census region, sociodemographic characteristics (median income and percent college graduates in the ZIP code of residence), and health status (CCI and PDG)) to determine whether engagement with the tool was associated with direct medical spending.

All analyses were conducted using Stata version 14. Written informed consent was received from all employees and the study was reviewed and approved by the New England Institutional Board #11-340.

## 3 Results

Of the approximately 11,000 employees nationwide we found 4220 employees who had used the commitment program in 2011, approximately a 38% participation rate. Administrative claims and enrollment data (including non-users of medical care services) was available from two main health plans offered by the employer for 6809 employees. Of these employees, 2124 had both commitment and claims data, a 31.2% participation rate (2124/6809) in the subset of employees where administrative claims data was available.

Employees made 25,716 commitments in 2011. The top commitments made by employees with the most frequent commitments falling into the categories Exercise Regularly (2477 commitments), and Annual HRA Questionnaire (2243). The third most frequent commitment, ‘Sleep Schedule,’ (1771) is not commonly addressed in most health promotion programs. The fourth highest is Lose Weight (1667) and the fifth highest is ‘Take My Meds,’ (1619) also not a typical component of a health promotion program, but is more in line with secondary prevention or disease management. Rounding out the top 10 commitments were: Maintain Weight (1406), Eat More of… (1331), Take a Lunch Break (1199), Cut That Out! (1165) and Moderate My Alcohol Consumption (1155). While the number of commitments made reflects repeated commitments by employees, the commitment made by greatest number of employees, was ‘Filled out the annual HRA questionnaire.’

Measures of engagement with health promotion activities as reflected by the tool included on average 5.65 (s.d. 9.64) commitments per employee in the first year, 0.39 referees (s.d. 0.43) per commitment, 0.61 supporters (s.d. 2.18) per commitment, and 11.71 journal entries (s.d. 58.69) per commitment. One third (33.3%) of employees completed the HRA and 15.2% completed the biometric screening. Employees earned an average of 2379.2 points (s.d. 5278.3) in the first year, 5.7% successfully completed a quit smoking commitment and 20% successfully completed a weight loss commitment.

Based on an analysis of the internal consistency of the program engagement measures we found that the logon user measure did not fit well with the other items (item-rest correlation was 0.019 and item-test correlation was 0.148 with all items exceeding 0.49). Without this item, average inter-item correlation was 0.434 and reliability analysis confirmed good consistency of these items (Chronbach's alpha = 0.894) representing engagement in online health promotion activities.

Combining the items using regression-derived factor scores yielded the following regression-derived item weights: use of the online commitment program (0.5897), number of commitments made (0.8770), percentage of commitments where the employee involved a referee for verification (0.7510), number of online supporters recruited by the employee (0.6016), number of online journal entries (0.5078), percent of reports made per commitment (0.8008), completion of the Health Risk Appraisal (HRA) (0.6582), completion of biometric screening (e.g., blood glucose and Body Mass Index) (0.6300), total points earned in 2011 (0.8627), completion of a quit smoking commitment (0.4124), and successful completion of a weight loss commitment (0.6534).

Table 2 displays the results of the analysis regressing the 2011 engagement index (calculated as a sum of standardized items) on employee characteristics, health status and 2010 spending and predicting the level of engagement. Employees age 18–34 had the highest rates of engagement with health promotion activities as reflected in the online commitment program and employees age 45 and over demonstrate the lowest engagement rates. Males age 18–34 had the highest level of engagement, and males age 55–64 had the lowest level of engagement overall. After adjusting for all other covariates, health status and 2010 spending had no association with engagement. Similar results were obtained using the weighted sum of standardized items as an engagement index.

**Table 2:** Regression of engagement index on sociodemographic characteristics and 2010 spending.

Variable	Coefficient	se	*p*
Age group (ref = 18–34 year old male)			
35–44 male	− 0.602	0.094	< 0.01
45–54 male	− 0.869	0.093	< 0.01
55–64 male	− 0.968	0.113	< 0.01
18–34 female	− 0.412	0.108	< 0.01
35–44 female	− 0.598	0.092	< 0.01
45–54 female	− 0.788	0.089	< 0.01
55–64 female	− 0.872	0.102	< 0.01
Urban residence	0.028	0.147	0.847
Region (ref = North East)			
North Central	0.169	0.077	0.027
South	− 0.125	0.073	0.086
West	− 0.121	0.110	0.275
Unknown	− 0.550	0.516	0.287
HMO	−0.052	0.043	0.228
Median household income in 3-digit zip	0.000	0.000	0.192
Percent college graduates in 3-digit zip	− 0.110	0.204	0.590
Deyo Charlson Comorbidity Index	0.008	0.034	0.810
Number of psychiatric diagnostic groupings	0.047	0.038	0.217
Total healthcare costs in 2010	0.000	0.000	0.419
Constant	1.524	0.190	< 0.01

Notes: 2011 Engagement with health promotion activities via an online health and wellness program by employees of a US large manufacturer.

Engagement index is a sum of the standardized items.

Adjusted R^2^ – 0.0679, Prob > F < 0.01.

Average 2011 spending in the sample was $6809 per employee per year. The association between 2011 total direct medical spending and the equal-weighted engagement index was negative (*p* = 0.153) (not shown), and the association between 2011 total direct medical spending and the weighted index using regression derived weights showed a 5.9% decrease in spending with a 1 standard deviation increase in the engagement index (*p* = 0.066).

## 4 Discussion

As employers look for ways to control costs of health care, they are increasingly looking for ways to engage employees in improving their health. Using data from a single employer, we found that the use of an incentive-based commitment program was used by a broad variety of employees within a single firm. Employees of all ages participated including those with a significant chronic illness or high levels of medical spending in the previous year. The program had the greatest use by younger employees. Engagement in health promotion activities was higher for younger (age 44 and under) and lower for older employees (above age 45), and engagement declined on a gradient with age. Perhaps engagement declined with age due to the online nature of the program, or perhaps the financial incentives were not large enough to attract older workers who have higher income on average.

When comparing the coefficients within each age group by gender, we do not see a consistent pattern. We found that older women (age 55–64) have a higher engagement rate than older men, but younger women (age 18–34) have a lower rate of engagement than younger men. This suggests that engagement with the health promotion tool is not uniform across demographic categories, and specific efforts to improve engagement may need to be developed to address each of the age and gender subgroups.

While it has been shown in previous research that employers should target employees at risk for chronic illnesses like diabetes, high blood pressure and high cholesterol levels, health promotion programs have tended to reach healthier employees. Our results show that participation, utilization, and engagement rates were no different between employees with and without a comorbidity, and those with high spending in the previous year also engaged with the commitment program. Perhaps the flexible aspect of the program to set their own health goals had greater appeal to those in need of secondary prevention and disease management.

The employees we studied comprise a mix of office-based and home-office (“field-based”) employees. Those in an office setting may have engaged with the program in different ways than field-based employees, as the wellness and engagement program was also promoted locally within the office setting via signs and local events including access to onsite biometric screening. As such, our findings represent an average effect of office and field-based employees. We did not have access to employee characteristics such as office/field-based, or other occupational characteristics, and those characteristics are important to collect and address in future studies.

We intend this study to be considered as a pilot of the concepts presented and not a formal test of reliability and validity. We believe that the measures we used are reliable to the extent that they are entered into the system by users. Several types of validity are addressed, although not formally tested, in our study: face validity that the measures reflect engagement, construct validity in the construction of measures and commitments within the system by experts in health and wellness, and criterion-related validity in the weakly negative correlation between engagement and 2011 spending.

While we used a unidemensional measure of engagement in health promotion activities, future studies could extend this exploration and determine the existence of multiple dimensions of engagement. We created two versions of the engagement index for comparison, one with equal weights for each item and a second using regression-based weight for each item. Results were largely consistent between the two indices, although the negative correlation between the index and future total direct medical spending was negative and not statistically significant for the equal-weighted index and was negative and weakly significant for the unequal-weighted index. This result also suggests future refinement of the construction of an online health promotion engagement index. Future studies could explore the relationship between the level of engagement in the program and following years' healthcare spending, which would yield valuable information about subsequent spending patterns. Also, future studies could examine whether the program yields long-term or short-term changes in behavior, and the sustained nature of these changes.

Many other patterns of use could be examined including: whether the type of activities employees are engaged in vary by age, by gender, by health status, by income, and by healthcare costs. Are there clusters of activity categories that people tend to engage? Are employees engaging more in areas which need the most attention or in the areas where they are already doing well? Finally, does familiarity of online system use play a role in engagement in health promotion activities?

Although higher levels of engagement with health promotion activities as reflected in the online program were associated with lower levels of spending this finding is not causal. Future studies should investigate the relationship between engagement and spending trends over a longer period of time using experimental and quasi-experimental methods.

## 5 Conclusions

Many US employers have been extending the size and scope of their incentive-based wellness programs since the final ruling on wellness incentives under HIPAA was released in 2006. Under these programs employers can provide incentives up to 20% of the value of the premium for outcome-based programs, those where the participant must meet a health target (e.g., cholesterol level) to receive the incentive. Progress-based programs, those without a specific health goal, where progress toward a health goal can have even higher levels of incentives. Health care reform amends qualifying wellness plan requirements as of 2014 and employers are permitted to allow a 30% incentive for achieving a health goal. In the NBGH survey of larger firms, 22% of employers are using outcomes-based incentives and two-thirds are using wellness incentives (LDI Issue Brief, 2011). While traditional wellness programs are likely to continue to be offered, comprehensive benefit design should consider participation-based programs as one way to reach the broader workforce. As a greater number of firms adopt incentive-based wellness programs additional research on utilization patterns and the impact of these programs will be important and necessary.

## Transparency document

Transparency document Image 1

## Conflict of interest statement

This work was supported by Bristol-Myers Squibb. All opinions expressed are those of the authors. Dr. Maclean reports that he held equity in BMS at the time the study was conducted.
